# Acute-Phase Serum Amyloid A in Osteoarthritis: Regulatory Mechanism and Proinflammatory Properties

**DOI:** 10.1371/journal.pone.0066769

**Published:** 2013-06-12

**Authors:** Dominique de Seny, Gaël Cobraiville, Edith Charlier, Sophie Neuville, Nathalie Esser, Denis Malaise, Olivier Malaise, Florence Quesada Calvo, Biserka Relic, Michel G. Malaise

**Affiliations:** 1 Laboratory of Rheumatology, GIGA Research, University of Liège, CHU Liège, Liège, Belgium; 2 Virology and Immunology Unit, GIGA Research, University of Liège, Liège, Belgium; INSERM-Université Paris-Sud, France

## Abstract

**Objective:**

To determine if serum amyloid A (A-SAA) could be detected in human osteoarthritic (OA) joints and further clarify if high A-SAA level in joints result from a local production or from a diffusion process from abnormally elevated plasma concentration. Regulatory mechanism of A-SAA expression and its pro-inflammatory properties were also investigated.

**Methods:**

A-SAA levels in serum and synovial fluid of OA (n = 29) and rheumatoid arthritis (RA) (n = 27) patients were measured and compared to matched-healthy volunteers (HV) (n = 35). *In vitro* cell cultures were performed on primary joint cells provided from osteoarthritis patients. Regulatory mechanisms were studied using Western-blotting, ELISA and lentiviral transfections.

**Results:**

A-SAA was statistically increased in OA plasma patients compared to HV. Moreover, A-SAA level in OA plasma and synovial fluid increased with the Kellgren & Lauwrence grade. For all OA and RA patients, A-SAA plasma level was higher and highly correlated with its corresponding level in the synovial fluid, therefore supporting that A-SAA was mainly due to the passive diffusion process from blood into the joint cavity. However, A-SAA expression was also observed *in vitro* under corticosteroid treatment and/or under IL-1beta stimuli. A-SAA expression was down-regulated by PPAR-γ agonists (genistein and rosiglitazone) and up-regulated by TGF-β1 through Alk1 (Smad1/5) pathway. RhSAA induced proinflammatory cytokines (IL-6, IL-8, GRO-α and MCP-1) and metalloproteinases (MMP-1, MMP-3 and MMP-13) expression in FLS and chondrocytes, which expression was downregulated by TAK242, a specific TLR4 inhibitor.

**Conclusion:**

Systemic or local A-SAA expression inside OA joint cavity may play a key role in inflammatory process seen in osteoarthritis, which could be counteracted by TLR4 inhibition.

## Introduction

Osteoarthritis (OA) is a degenerative disorder characterized by a progressive cartilage breakdown, osteophyte formation, subchondral bone thickening and local inflammatory process. It is now considered as a metabolic disorder since mechanical stress alone cannot explain the link between obesity and pathology in non-weight-bearing joints, such as hand OA [1]–[4].

Recent evidences suggest that chondrocytes can under physiological and pathological conditions synthesize several non-matrix factors like adipokines that contribute to cartilage degradation within articular joints [5]. As observed in rheumatoid arthritis (RA), fibroblast-like synoviocytes (FLS) might play a role in joint destruction by producing cytokines and metalloproteinases [6]. FLS are also able to secrete adipokines such as leptin [7].

Leptin, adiponectin, resistin and visfatin are the most extensively studied adipokines in OA [5]. However, A-SAA (SAA1 and SAA2, collectively called A-SAA) has been recently placed on the front stage of research for its convergence to both inflammation and metabolic pathways [8], [9].

A-SAA is highly produced by the liver after stimulation with pro-inflammatory cytokines. Its concentration may increase up to 1000-fold during the acute phase of inflammation in regard to normal condition [10], [11]. Besides its influence on lipid metabolism [12], [13], A-SAA is known to participate to immune cells recruitment at inflammatory sites [14], [15] and to induce expression of pro-inflammatory cytokines [14], [16] and matrix metalloproteinases [17]. Across the last decade, several studies attempted to demonstrate the extra-hepatic production of A-SAA by several tissues and different cell types of patients with atherosclerosis [18], Alzheimer disease [19], obesity [9] or RA [20], [21]. A-SAA protein was detected in synovial membrane provided by RA patients as well as in RA-synoviocytes [20]. A-SAA protein was similarly detected in the synovial membrane of patients with psoriatic arthritis, sarcoïdosis or other undifferentiated arthritidies [20]–[22] and slightly detected in sections from paraffin-embedded OA cartilage [23].

A-SAA mRNA was found to be up-regulated by TNF-α, IL-1β and to a lesser extent by IL-6 in FLS of RA patients [20]. FLS and chondrocytes capacity of secreting A-SAA at a protein level is largely unknown. Indeed, data are still inconsistent and even conflicting in determining if high A-SAA level in joints are due, at least in part, to a high local production in pathological tissues or if the A-SAA diffusion into pathological tissues largely depends on its plasma concentration [14]. Furthermore, molecular and cellular mechanisms related to A-SAA expression by extra-hepatic cells are poorly understood. Therefore, to further clarify the role played by A-SAA as a systemic or local inflammatory marker, A-SAA production was studied *in vitro* using human chondrocytes, FLS and preadipocytes.

## Patients and Methods

### Patients

Twenty-nine patients with OA and 27 with RA recruited through community questionnaires, consultations and hospital outpatient clinics took part in this study. All the patients fulfilled the established diagnostic criteria for their respective diseases [24], [25]. OA patients were classified according to the Kellgren and Lawrence grade (K&L) [26]. None of the OA patients were on intra-articular steroid. Among the 27 RA patients, some of them received concomitant treatments including methotrexate (MTX) at a mean dosage of 9.4 mg/week (range 6–15 mg/week) in 11 (40%) of 27 patients and prednisolone at a mean dosage of 8.9 mg/day (range 5–20 mg/day) in 15 (55%) of 27 patients. Thirty-five healthy volunteers (HV), matched for age [median (range) yr: 56 (50–64)], gender (56% of female) and BMI [median (range) Kg/m^2^: 25 (19–31)] served as the control group. HV were qualified for entry into the study considering the following exclusion criteria: any prior history of knee trauma, joint pain, chronic inflammatory or autoimmune disease, cardiovascular disease, diabetes, corticosteroid injection or NSAID use. Paired synovial fluid (SF) and blood samples were simultaneously collected for each patient.

### Ethics Statement

The institutional review boards (Research Ethics Committee) of the University Hospital, CHU de Liège, Belgium, approved both the study protocol and the use of verbal informed consent to allow research procedures on biologic samples or tissues collected in out- and in-patients during their passage or stay at the CHU de Liège, as explained in the institutional information booklet written by the hospital and provided to each patient. Clinicians gave to patients an oral statement about our research. This statement included basic elements of our informed consent. Then, clinicians informed patients about authorizations obtained from the local Research Ethics Committee to perform studies and showed documents to those who wanted to read it. Clinicians gave sufficient time to patients to consider whether or not they wanted to participate in the research. After allowing the potential subject sufficient time, clinicians answered to any additional questions patients might have. Then clinicians obtained patient’s verbal consent to participate in the research. Strict data management retained anonymity of participants.

### Human primary chondrocytes, FLS and preadipocytes

The cartilage and synovial tissue were obtained from OA patients during joint replacement. Informed consents were obtained and experiments approved by the ethics committee of our academic hospital (CHU, Liège, Belgium). Primary chondrocytes and FLS were isolated from human cartilage and synovial tissue, respectively, as described in [27] [28]. Briefly, primary chondrocytes (2×10^5^ cells/0.5 ml of medium) were seeded in 24-well plates (BD Biosciences, USA) in triplicate and let to dedifferentiate or stimulated with indicated reagents at indicated concentration and defined culture time. FLS (5×10^4^ cells/0.5 ml of medium) were seeded similarly between passages 2–6. After a defined culture time, cellular supernatants were collected for ELISA tests and cells were harvested for protein extraction.

Visceral adipose tissue samples were minced and digested with 1.5 mg/ml type I collagenase (Sigma-Aldrich, USA) in PBS for 90 min at 37°C with manually shaking for 5–10 seconds every 15 min. To stop collagenase activity, FBS (10%) was added. After centrifugation (400 *g* for 10 min), the floating mature adipocytes were removed and the stroma vascular fraction (SVF) cell pellet was resuspended in erythrocyte lysis buffer (155 mmol/l NH_4_Cl; 5.7 mmol/l K_2_HPO_4_; 0.1 mmol/l EDTA; pH 7.3) for 10 minutes. After successive washes and a filtration through a 70 µm nylon cell strainer (BD Biosciences, USA), the SVF cells were suspended in PBS supplemented with 3% FBS before being stained for flow cytometry. A part of the SVF cells were stained with saturing amounts of various fluorochrome-conjugated antibodies such as CD45- phycoerythrin(PE)- cyanin 5 (PC5), CD14-fluorescein isothiocyanate (FITC), CD3- HorizonTM V500, CD4-efluor 450, CD8- Perdidin chlorophyll protein (PerCP), CD25- phycoerythrin (PE), CD127 biotin, streptavidin allophycocyanin (APC)- efluor 780 (4°C, 20 min, dark). All antibodies were obtained from Ebioscience, with the exception of CD3-HorizonTM v500, CD8-PercP and CD14-FITC (BD Biosciences, USA). Flow cytometry was performed on a FACSAria (BD Biosciences, USA). Data were acquired, analyzed and plotted using FacsDiva 6.1.2 (BD Biosciences, USA). The subpopulations of SVF were determined based on sideward scatter, CD45 and CD14 expression. Preadipocytes were sorted from the CD45^-^CD14^low^ stromovascular populations with a purity of > 90% in all samples as described in [29]. Cells (5×10^4^ cells/0.5 ml of medium) were seeded in 24-well plates as described for FLS.

### Culture reagents

Culture reagents were purchased as follows: recombinant human SAA (rhSAA, #300-13, Peprotech, USA), TNF-α (Biosource, Belgium), IL-1β, IL-6 (R&D systems, USA), rosiglitazone, genistein (Alexis Corp., USA), leptin, dexamethasone, prednisolone, mifepristone, eplerenone, spironolactone, aldosterone, SB431542, lipopolysaccharides (LPS), phenylmethanesulfonyl fluoride (PMSF) (Sigma-Aldrich, USA), insulin (Humulin, Lilly, France), transforming growth factor β1 (TGF-β1) (GIBCO-BRL, USA), compound A (CpdA), IgG rabbit polyclonal control, IgG1 mouse monoclonal (Santa Cruz Biotechnology, USA), anti-RAGE IgG rabbit polyclonal (Abcam, UK, #ab37647), anti-CLA1/SR-B1 (BD Biosciences, USA, #610883), lipoxin A4 (Cayman Chemical, Bioconnect, NL), TAK242 (CLI-095, InvivoGen, USA), sulfo-N-succinimidyl oleate (SSO, Santa Cruz Biotechnology USA) and polymyxin B (Calbiochem Novabiochem, USA).

### ELISA

A commercially available sandwich enzyme-linked immunosorbent assay was used for IL-6, IL-8, GRO-α, MCP-1, MMP-1, MMP-3 and MMP-13 (R&D Systems, USA) and A-SAA (Invitrogen, USA) quantification in serum/plasma, synovial fluid and in cell supernatants according to manufacturer’s instructions. RayBio Human cytokines/MMP antibody arrays were performed according to manufacturer’s instructions (RayBiotech, USA).

### Western Blot

Cells were collected, lysed with RIPA buffer (150 mM NaCl, 1% NP40, 0.5% deoxycholate, complete, 0.1% SDS and 50 mM Hepes pH 7.5) and total proteins separated by SDS-PAGE as explained earlier in [28]. Membranes were incubated with A-SAA (Abcam, UK), p-Smad2 (S465/467), Smad2 (L16D3), p-Smad1/5 (S463/465), Smad1 (Cell Signaling Technology, USA) or GAPDH (Sigma-aldrich, USA) antibodies overnight. Western blots were visualized with 1∶2000 diluted anti-mouse or anti-rabbit (DAKO A/S, Denmark) antibodies and ECL chemiluminescent reagents (GE Healthcare, UK).

### Endotoxin contamination

Endotoxin level in rhSAA was indicated as below 0.1 ng/µg according to manufacturer’s instructions. 0.1 ng/µg endotoxin level is equivalent to 0.5 ng/mL (or 5EU/mL) endotoxin level when 5 µg of rhSAA is supplied to 1 mL of *in vitro* culture medium. To further quantify the endotoxin level of our used solution, 10 µg rhSAA were diluted in 1 mL PBS and sent for analysis to Lonza Company (Verviers, Belgium). They reported an endotoxin level below to 5 pg/mL (or 0.05 EU/mL).

Polymyxin (0.2 µg/mL) or proteinase K (10 µg/mL) was first incubated with rhSAA or LPS during 45min at 37°C. PMSF (10 µM) was then added to proteinase K/rhSAA solution for next 45min at 37°C for degrading proteinase K enzyme (a cytotoxic protease) before cell incubation.

### Transfection with lentiviruses expressing Smad1 shRNA

Lentiviral vectors were generated by co-transfecting Lenti-X 293T cells (Clonetech, Belgium) with a pSPAX2 plasmid (Addgene, Plasmid #12260), a VSV-G encoding vector [30] and a Smad1 shRNA plasmid (#TRCN0000423087 (shRNA1), #TRCN0000021780 (shRNA2) or #TRCN0000432716 (shRNA3), Sigma-Aldrich, USA) or with a non target sequence encoding plasmid (Sigma, Belgium, SHC002). Seventy-two hours post transfection, viral supernatants were collected, filtrated and 100X concentrated by ultracentrifugation. Lentiviral vectors were then titrated using qPCR Lentivirus Titration(Titer) Kit (ABM, USA, LV900). Primary human chondrocytes and FLS were plated at a density of 2×10^5^ cells/0.5 mL in DMEM supplemented with 10% FBS in quadruplicate in 24-well plates. After 7 days of culture, medium was replaced and cells were infected by lentivirus at MOI 100. After 72h incubation, cells were stimulated with prednisolone (1 µM) during 48h. Supernatants and cell lysates were collected for ELISA test (A-SAA) and Western blotting (Smad1 and GAPDH), respectively.

### Statistical analysis

Statistical analysis was performed by GraphPad Prism software. A-SAA levels in plasma and SF were compared using the nonparametric Mann-Whitney Test and were expressed as median and interquartile range. Other graphs represent averages of triplicates ± SD from one representative of three independent experiments. Correlation coefficients were calculated using Spearman test. p Values <0.05 were considered statistically significant.

## Results

### A-SAA levels in plasma and synovial fluid

**Figure 1:**
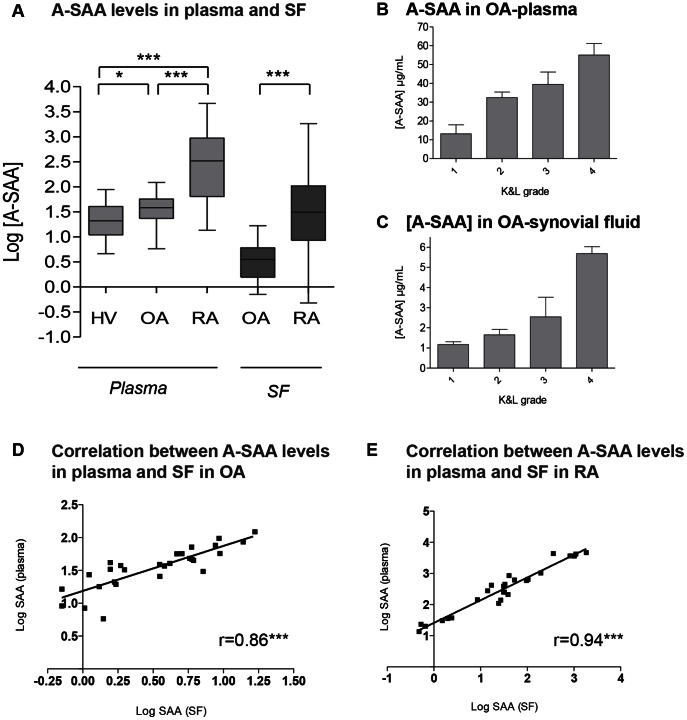
A-SAA levels in plasma and synovial fluid – A-SAA according to K&L grades in OA. (A) A-SAA profiles in blood and synovial fluid (SF) of OA and RA patients compared to those of matched healthy volunteers (HV). The boxes illustrate the 25th-75th percentiles, the horizontal lines the medians and the vertical lines the minimum and the maximum values. *p<0.05, **p<0.01 and ***p<0.001; non parametrical Mann-Whitney test. A-SAA expression level in (B) plasma and in (C) synovial fluid (SF) of OA patients classified according to their Kellgren and Lauwrence (K&L) grades. Correlation of A-SAA levels in plasma vs. A-SAA levels in SF for OA (D) and RA (E) patients.

Plasma A-SAA (Figure 1A) levels were significantly higher in the OA than in the HV group and remained lower in the OA than in the RA group. A-SAA was easily detected in the synovial fluid of OA patients but levels remained significantly lower compared to synovial levels of RA patients. For all OA and RA patients, A-SAA plasma level was higher compared to its corresponding level in the synovial fluid in a range of 3 to 44 fold. Plasma A-SAA levels were significantly correlated to their synovial fluid levels in both OA and RA patients (Figure 1D–E). A-SAA expression level increased in both plasma (Figure 1B) and synovial fluid (Figure 1C) according to the severity of OA, which was characterized by Kellgren & Lauwrence grades [26].

### A-SAA secretion by human OA joint cells and preadipocytes

#### Chondrocytes and cartilage shavings

**Figure 2:**
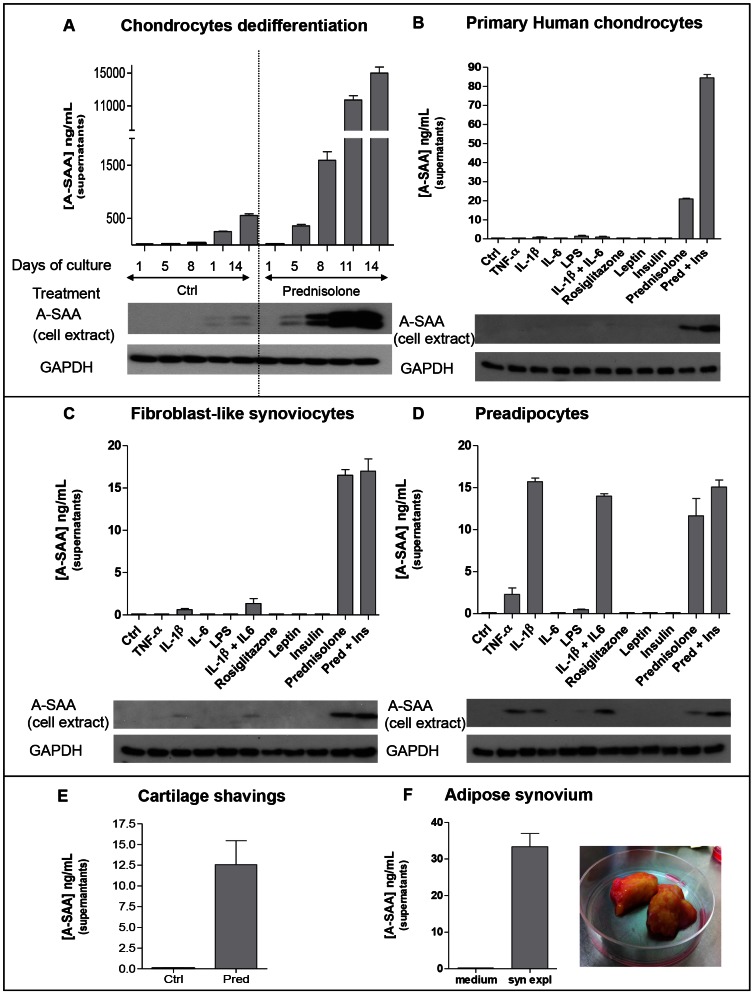
A-SAA secretion by several cell types and tissues originated from OA patients. Detection of A-SAA secreted by (A) chondrocytes along dedifferentiation process, (B) primary human chondrocytes after 48h of treatment, (C) fibroblast-like synoviocytes after 1 week of treatment, (D) preadipocytes after 1 week of treatment with TNF-α (10 ng/mL), IL-1β (10 ng/mL), IL-6 (10 ng/mL), LPS (10 ng/mL), rosiglitazone (10 µM), leptin (100 nM), insulin (100 nM) and prednisolone (1 µM), (E) Secretion of A-SAA by cartilage shavings (0.2 g) after 1 week of treatment with prednisolone (1 µM). (F) Spontaneous secretion of A-SAA by fat synovial membrane after 48h in culture cell medium. A-SAA was determined in the culture supernatants (ELISA) and in cell extracts (Western blotting).

Human primary chondrocytes when cultured during 14 days in monolayer undergo cell shape changes, shifting from round form to flattened form, a process known as dedifferentiation [31], [32]. Here we observed that A-SAA secretion by chondrocytes increased with dedifferentiation progression, and was even markedly enhanced under prednisolone (Figure 2A). Prednisolone also enhanced A-SAA secretion by cartilage shavings (Figure 2E) and primary chondrocytes (still round shaped, only cultured 48h), an effect reinforced by insuline (Figure 2B).

#### Fibroblast-like synoviocytes

FLS were cultured during 48h, 72h and 1 week. However, prednisolone-induced A-SAA expression was only detected after one week of treatment (Figure 2C). A-SAA expression was also slightly observed in the presence of IL-1β treatment (Figure 2C).

#### Visceral preadipocytes and adipose synovium

Visceral preadipocytes were used as extra-articular cells known as good responders to inflammatory stimuli for inducing A-SAA expression. Indeed, we observed that A-SAA protein was slightly detected under TNF-α or LPS but further enhanced under IL-1β or prednisolone. Cells remained insensitive to rosiglitazone, leptin or insulin (Figure 2D). Spontaneous secretion of A-SAA was observed with adipose synovium explants (Figure 2F).

### Glucocorticoid-induced A-SAA expression is GR-dependent and downregulated in the presence of PPAR-γ agonists

**Figure 3:**
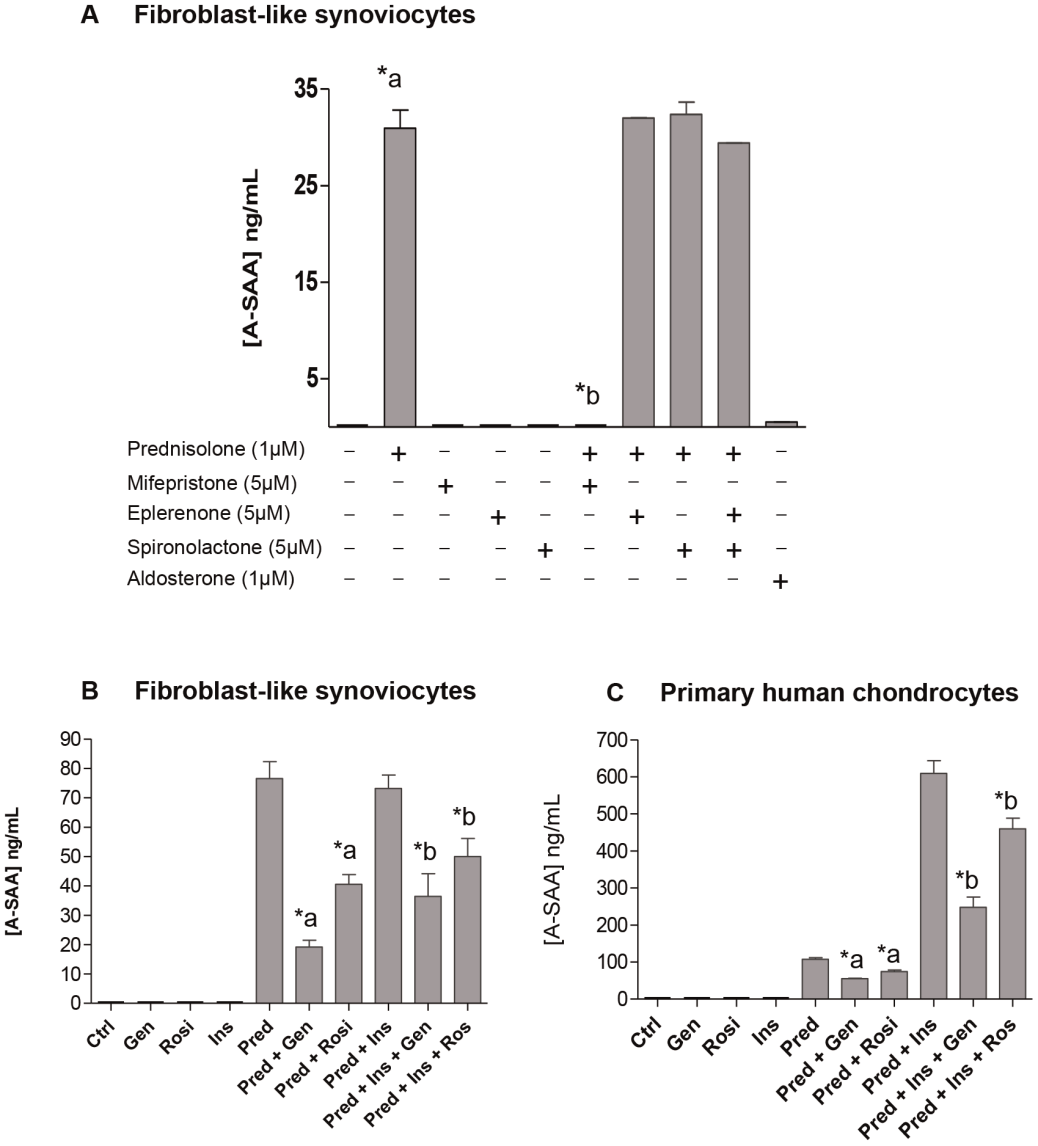
Glucocorticoid-induced A-SAA expression is GR-dependent and downregulated in the presence of PPAR-γ agonists. (A) OA-fibroblast-like synoviocytes (FLS) were stimulated during 1 week with prednisolone (1 µM), mifepristone (5 µM), eplerenone (5 µM), spironolactone (5 µM) and aldosterone (1 µM). Prednisolone-induced A-SAA expression was inhibited by the GR inhibitor mifepristone but not by MR inhibitors (eplerenone and spironolactone). A-SAA expression was barely detectable under aldosterone treatment, a MR-dependent glucocorticoid. *a, statistically different from control. *b, statistically different from prednisolone-treated cells. A-SAA expression level by (B) FLS after 1 week and (C) primary human chondrocytes after 72h (A) of treatment with prednisolone (1 µM), genistein (50 µM), rosiglitazone (10 µM) and/or insulin (100 nM). *a, statistically different from prednisolone-treated cells; *b statistically different from prednisolone- and insulin-treated cells.

To know the involvement of both glucocorticoid- (GR) and mineralocorticoid- (MR) receptors in glucocorticoid-induced A-SAA expression, FLS were stimulated with prednisolone (GR-dependent glucocorticoid), aldosterone (MR-dependent glucocorticoid) and GR inhibitor (mifepristone) or MR inhibitors (eplerenone and spironolactone). A-SAA expression level was increased under prednisolone but not using the MR-dependent aldosterone (Figure 3A). Furthermore, prednisolone-induced A-SAA expression was inhibited by the GR inhibitor mifepristone but not by MR inhibitors (eplerenone and spironoloactone) (Figure 3A). These results suggested that glucocorticoid-induced A-SAA expression is GR- but not MR-dependent. Similar results were observed with chondrocytes (data not shown).

FLS and primary human chondrocytes were then treated with peroxisome proliferators-activated receptor gamma PPAR-**γ** agonists (genistein and rosiglitazone) with or without prednisolone and/or insuline. A-SAA expression was induced in both cell types under prednisolone treatment. Both genistein and rosiglitazone downregulated A-SAA production level when both cell types were stimulated with prednisolone alone or combined with insulin (Figure 3B and 3C). These results showed that A-SAA expression could be downregulated *via* the PPAR-γ pathway.

### Inhibition of A-SAA expression by TGF-β1 in human primary chondrocytes and FLS

**Figure 4:**
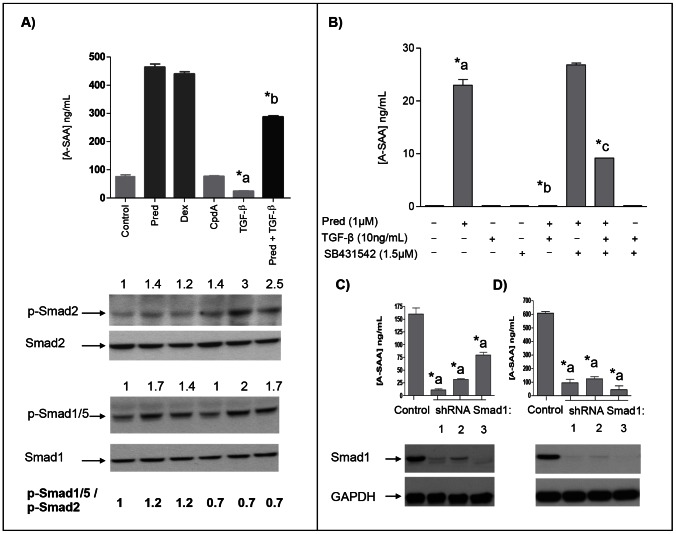
Balance through either p-Smad2 or p-Smad1/5 signalling. (A) Human chondrocytes were stimulated at day 7 for 48h in the presence or absence of prednisolone (1 µM), dexamethasone (1 µM), compound A (CpdA) (1 µM) and/or TGF-β (10 ng/mL). A-SAA was determined by ELISA in the culture supernatants. p-Smad2, Smad2, p-Smad1/5 and Smad1 expression were detected by western blotting in cell extracts. Quantification was performed using ImageQuant LAS 4000 software. Ratio [p-Smad1/5]/[p-Smad2] is also indicated. (B) Primary human chondrocytes were stimulated at day 1 during 72h in the presence or absence of prednisolone (1 µM), TGF-β (10 ng/mL) and SB431542 (1.5 µM). A-SAA (ELISA) and Smad1 expression (Western blotting) in dedifferentiated chondrocytes (C) and FLS (D) transduced with lentiviral particles for shRNA control or three different shRNA of Smad1. *a, statistically different from control. *b, statistically different from prednisolone-treated cells. *c, statistically different from prednisolone and TGF-β treated cells.

We have demonstrated that glucocorticoids (prednisolone and dexamethasone) but not compound A (CpdA; a non-steroidal GR modulator) significantly increased A-SAA production level in human dedifferentiated chondrocytes (Figure 4A) and in FLS (data not shown).

We next investigated the role of TGF-β1 in A-SAA expression. Endogenous- and glucocorticoid-induced A-SAA expressions in chondrocytes were significantly decreased when cells were stimulated at day 7 during 48h with TGF-β1 (Figure 4A). Prednisolone-induced A-SAA expression was even completely abolished by TGF-β1 when primary chondrocytes were stimulated earlier at day 1 (Figure 4B).

As TGF-β1 is known to signal via Alk1 (Smad1/5 pathway) and/or Alk5 (Smad2/3 pathways) [33], [34], both pathways were investigated by three different approaches. First, semi-quantitative level of p-Smad2, Smad2, p-Smad1/5 and Smad1 were obtained by Western blotting. Accordingly, it was observed that glucocorticoids increased Smad1/5 phosphorylation [p-Smad1/5] and had slight effect on Smad2, thereby favouring Alk1-Smad1/5 pathway as indicated with the positive quantitative [p-Smad 1/5]/p-Smad2 ratio of 1.2 (Figure 4A). On the other hand, TGF-β1 also induced Smad1/5 phosphorylation but favoured Alk5-Smad2 pathway by increasing Smad2 phosphorylation ([p-Smad 1/5]/p-Smad2 ratio of 0.7), counteracting by the way Alk1-Smad1/5 pathway. Secondly, SB431542, an Alk5-Smad2 inhibitor, partially restored TGF-β1 inhibition of a previous prednisolone-induced A-SAA expression (Figure 4B). Finally, lentiviral particles expressing three different Smad1 shRNA were used to infect dedifferentiated chondrocytes and FLS. Western blot analysis demonstrated a significant decrease of Smad1 expression in infected chondrocytes (Figure 4C) and FLS (Figure 4D). ELISA analysis showed that Smad1 silencing resulted in a significant decrease in A-SAA expression (Figure 4C et 4D). These results suggest that Smad1 is positively involved in A-SAA expression level in both cell types.

### rhSAA-inducing cytokines and MMPs/TIMPs expression by human chondrocytes and FLS

Raybio human cytokine/MMP/TIMP protein arrays were used to screen cytokines or MMPs/TIMPs released in the medium by chondrocytes or FLS in the presence or absence of rhSAA (Figure S1). Of the 23 cytokines pairs on the array, cytokines up-expressed by chondrocytes and FLS in the presence of A-SAA were GRO, IL-8, IL-6 and MCP-1. Of the 10 MMPs/TIMPs pairs on the array, MMPs secreted by chondrocytes in the presence of A-SAA were MMP-1, MMP-3 and MMP-13. RhSAA-induced cytokines and MMPs identified by protein arrays were further validated by ELISA assays (Figure S2). Increasing concentrations of rhSAA were tested in the range of 0.5 µg/mL to 5 µg/mL. The effect of rhSAA on targeted cytokines/MMPs expression in chondrocytes and FLS was clearly dose dependent (Figure S2).

### TLR4 mediates A-SAA-induced cytokines and MMPs expression

**Figure 5:**
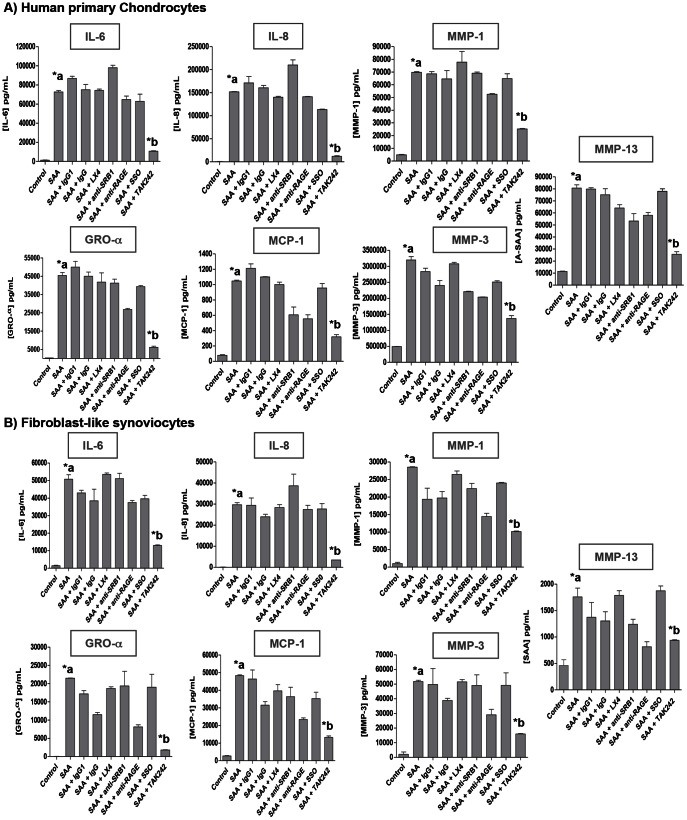
TLR4 mediates A-SAA-induced cytokines and MMPs. Blockade of rhSAA (2.5 µg/mL)-induced cytokines and MMPs by specific inhibitors: lipoxin A4 (LX4; 1 µM), anti-SR-B1 antibody (5 µg/mL) and its isotype IgG1 control antibody (5 µg/mL), anti-RAGE (10 µg/mL) and its isotype IgG control antibody (10 µg/mL) and TAK242 (1 µM) for FPRL1, SR-B1, RAGE and TLR4 recepetors, respectively, on A) OA primary chondrocytes and B) OA FLS after 24h of stimulation. Cytokines and MMPs levels were quantified by ELISA in the culture supernatants. *a, statistically different from control. *b, statistically different from rhSAA-treated cells.

A-SAA is known for interacting with several receptors including formyl peptide receptor like-1 (FPRL1) [21], [35], scavenger receptor class B member I (SR-B1/CLA1) [36], CD36 [37], toll-like receptors (TLR2 and TLR4) [38], [39] and receptor for advanced glycation end-products (RAGE) [40], [41]. Several inhibitors were used to investigate which receptor was involved in A-SAA-induced cytokines/MMPs secretion by chondrocytes (Figure 5A) and FLS (Figure 5B). We used LX4 known to compete with A-SAA for FPRL1 binding [14], [42], anti-RAGE or anti-SRB1 antibodies to block respective receptors, as well as SSO, which irreversibly inhibit CD36. None of these molecules were able to significantly diminished proinflammatory cytokines secretion (Figure 5A and B). However, using TAK242, SAA-induced cytokines and MMPs were significantly decreased in chondrocytes and FLS (Figure 5A and 5B). TAK242 (resatorvid) is a small molecule known for binding selectively the intracellular domain of TLR4 among 10 different human TLRs [43]. All together, these results showed that TLR4 could mediate SAA-induced cytokines and MMP, in both chondrocytes and FLS (Figure 5A and B). We further assessed the specificity of our results by demonstrating that rhSAA-induced IL-6 expression levels in chondrocytes and FLS were not due to endotoxin contamination.

### Specificity of the results obtained with rhSAA

**Figure 6:**
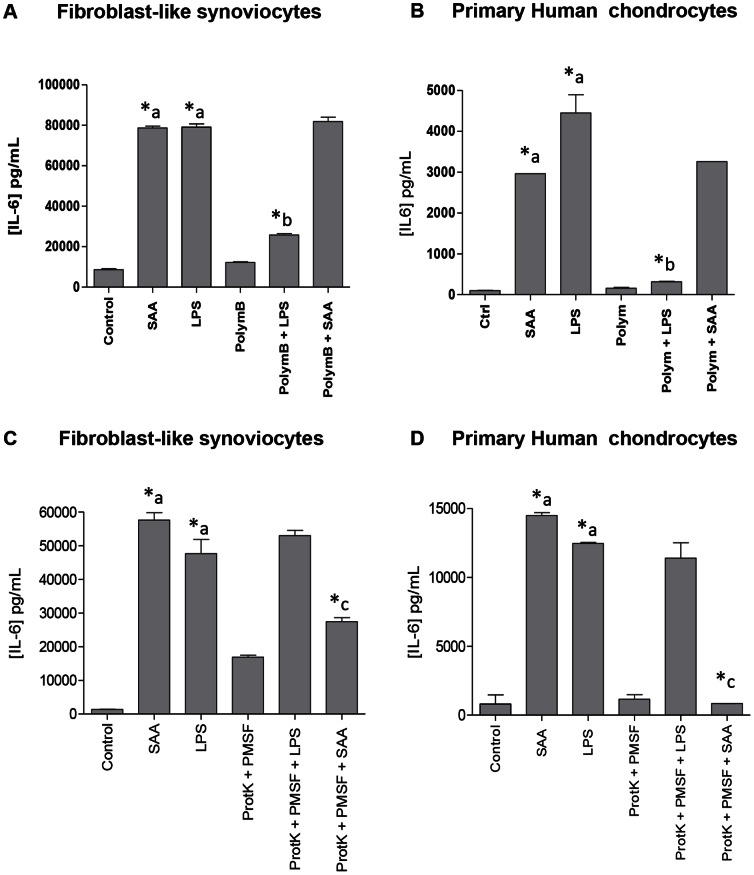
Endotoxin contamination - Polymyxin B and Proteinase K digestion treatment. OA fibroblast-like synoviocytes (A–C) and human primary chondrocytes at day 1 (B–D) were stimulated during 24h with rhSAA (5 µg/mL) and LPS (2 ng/mL) in the presence or not of A–B) polymyxin B (0.2 µg/mL) or C–D) Proteinase K (10 µg/mL) and PMSF (10 µM). IL-6 level was quantified by ELISA in the culture supernatants. *a, statistically different from control. *b, statistically different from LPS-induced cells. c*, statistically different from SAA-induced cells.

To rule out the possible LPS effect, we compared IL-6 production induced by rhSAA (5 µg/mL) with those obtained with LPS 2 ng/mL (20 EU/mL), last concentration being largely superior to the one detected in our *in vitro* experiments (<0.05 EU/mL according to Lonza – 5 EU/mL according to the supplier). PolymyxinB is a cationic cyclic lipopeptide that binds stoechiometrically to the lipid A moiety of LPS and blocks its biological effects, whereas proteinase K is a serine protease that cleaves peptide bonds at the carboxylic sides of amino acids degrading A-SAA protein. When rhSAA and LPS were both combined with polymyxinB, IL-6 induction was drastically decreased for PolymB/LPS but not for PolymB/rhSAA (Figure 6A–B). Conversely, proteinase K treatment inhibited IL-6 production by rhSAA but not by LPS (Figure 6C–D). These results suggest that rhSAA, but not LPS, was responsible to pro-inflammatory effects observed previously.

## Discussion

A-SAA level was statistically increased in OA plasma patients compared to HV. Moreover, A-SAA level in OA plasma and synovial fluid increased with the Kellgren & Lauwrence grade, a radiological score used to evaluate the severity of OA [26]. A-SAA level was statistically increased in RA plasma compared to those in HV and OA plasma as already described [44], [45]. For all OA and RA patients, A-SAA plasma level was higher compared to its corresponding level in the synovial fluid in a range of 3 to 44 fold. Furthermore, A-SAA plasma was highly correlated with its corresponding A-SAA-synovial fluid level for both OA and RA pathologies. These two observations were therefore in favour of a passive diffusion of A-SAA from circulating fluid to the synovial joint rather than the opposite. Nevertheless, endogenous A-SAA secretion was also investigated using *in vitro* studies on primary cells isolated from OA joints after surgical replacement.

Among cells tested, dedifferentiated chondrocytes and adipose synovial explants were the only ones able to spontaneously secrete A-SAA. Primary chondrocytes, when liberated from their extracellular matrix and cultured in a monolayer, shift from their typical spherical shape to polygonal flattened fibroblast-like shape, a process termed dedifferentiation [31], [32]. This morphological change is accompanied by changes in gene expression profile, from cartilage- specific genes (*e.g.* type –II, -IX, -XI collagen, aggrecan…) to genes that are rather expressed by fibroblasts (*e.g.* type –I, -III, -V collagen, versican…) [46]. Dedifferentiation constitutes a real problem in the field of articular cartilage repair as it avoids primary chondrocytes expansion *ex vivo* and subsequent re-implantation strategy, also termed autologous chondrocytes transplantation (ACT) [47]. However, the molecular mechanisms of signal transduction involved in this process remain poorly understood [31], [32], [48], [49]. As a consequence, it is of high interest to better characterize primary chondrocytes *vs* dedifferentiated chondrocytes at the molecular level.

Unexpectedly, we have observed that glucocorticoids but no other investigated mediators were able to induce A-SAA protein secretion by human primary cells from OA joints origin. Two prestigious publications have already described the following paradox of an anti-inflammatory drug (glucocorticoids) inducing the pro-inflammatory mediator (A-SAA). Bozinovski et *al.* [42] have described that glucocorticoids markedly increased SAA in human macrophages THP-1 cell line in chronic obstructive pulmonary disease models. Yang RZ *et al.* [9] have demonstrated that A-SAA expression was induced by human adipose tissue explants under dexamethasone stimulation. Of interest our data have shown a common stimulatory response to corticosteroid for all cell types but a restricted stimulatory response to cytokines indicating that A-SAA mechanisms of expression are cell-dependent: chondrocytes being unresponsive to pro-inflammatory cytokines, OA fibroblast-like synoviocytes poorly sensitive, and preadipocytes obtained from visceral tissue fully sensitive. A-SAA secretion from adipose synovium could therefore arise from preadipocytes under IL-1β-autocrine or -paracrine secretion by macrophages, adipocytes or FLS themselves.

Limited number of human cell types including KB oral epithelial cells [50], [51], aortic smooth muscle cells [52] and adipose tissue explants (35), have demonstrated their capacity of producing A-SAA under glucocorticoid alone, whereas hepatic cell such as Hep-3B [53] or HepG2 [50], [51] need a preliminary cytokine-driven induction of A-SAA before corticosteroid enhancement [50]–[52].

Obviously, these publications strengthened the urgent need to clarify glucocorticoid impact on protein expression level and their related physiological consequence, mainly in the rheumatology field where glucocorticoids are abundantly used as therapeutic treatment.

Thorn et *al.* had previously detected a putative glucocorticoid response element (GRE) functionally active in the SAA1 gene whereas it was disrupted in the SAA2 gene [51]. They had also described that GR was a required component in mediating the dexamethasone enhancement of cytokine driven SAA1 transcriptional activity [51] using HepG2 cells. Conde *et al.* also observed that dexamethasone was able to increase SAA3 mRNA expression in murine chondrogenic cell line ATDC-5 [54]. In our cells that spontaneously respond to corticosteroid exposure, we have shown that A-SAA secretion was strictly GR dependent as totally abrogated in the presence of mifepristone, whereas aldosterone antagonists (eplerenone and sprironolactone) had no effect on A-SAA production, excluding MR involvement.

Considering our previous work on FLS adipogenesis [7], cells were cultured in the presence of PPAR-γ agonists: genistein and rosiglitazone. Accordingly, we have observed that PPAR-γ agonists induced a significant inhibition of glucocorticoid-induced A-SAA production. Yang et *al.* had already described similar results on human adipose tissue [9]. They had observed that A-SAA expression increased in the presence of dexamethasone and/or insuline and was down-regulated with rosiglitazone. In our study, increased expression of glucocorticoid-induced A-SAA was observed in the presence of insulin but for chondrocytes cells only.

Recent studies performed in our laboratory have demonstrated the inhibitory effects of TGFβ1 on leptin expression in FLS and BM-MSC [55]. ALK1/ALK5 was proposed as a regulatory balance for leptin expression as already described for MMP13 [33]. In the present work, similar TGFβ1 inhibitory effects were observed for A-SAA protein expression on chondrocytes and FLS. ALK1/ALK5 was also proposed as a regulatory balance for A-SAA expression. The protective role of TGFβ1 would run through the ALK5 (Smad2/3) pathway whereas ALK1 (Smad1/5) pathway would further be considered as pro-inflammatory enhancing A-SAA expression. Our results strengthened the hypothesis developed by Blaney *et al*. for which a shift in balance toward ALK1 is observed contributing to age-related cartilage destruction and OA [33]. We support the concept that ALK1 rather than TGFβ1 could be a promising therapeutic target.

Besides A-SAA regulatory mechanisms, downstream effects of A-SAA on cytokines/MMPs expression by chondrocytes and FLS were assessed using rhSAA protein. Increased expression levels of IL-6, IL-8, GRO-α, MCP-1, MMP-1, MMP-3, and MMP13 were observed in a rhSAA-dose dependent manner. It is noteworthy to mention that rhSAA concentration used for our *in vitro* studies was similar to the one detected in the synovial fluid of our OA cohort. SAA-induced cytokines [IL-6 [16], [41], IL-8 [14] and MCP-1 [14]] or MMPs [MMP-1, MMP-3 [21] and MMP-13 [17]] or cell adhesion factors [ICAM/VCAM [15]] were already described in previous studies but performed on RA- but not OA-synoviocytes. It was also recently published that A-SAA increased MMP-1, MMP-3 and MMP-13 mRNA and protein expression levels on human chondrosarcoma cells SW1353 and human primary chondrocytes provided from normal articular cartilage [17]. However, we have clearly demonstrated that TAK242 was the only inhibitor able to block significantly A-SAA-inducing cytokines and MMPs expression in chondrocytes and FLS. It strongly suggests that A-SAA is an endogenous TLR4 agonist in OA as already observed by Sandri *et al.* in mouse macrophages [39]. Furthermore, it was recently published that sCD14 in synovial fluid of early OA patients increased IL-8 and IL-6 expression by FLS in response to both TLR2 and TLR4 ligands [56], therefore supporting the role of innate immunity in low-grade inflammation observed within the synovial membrane of OA joints. We further assessed the specificity of our results by demonstrating that rhSAA-induced IL-6 expression levels in chondrocytes and FLS was not due to endotoxin contamination.

## Conclusion

**Figure 7:**
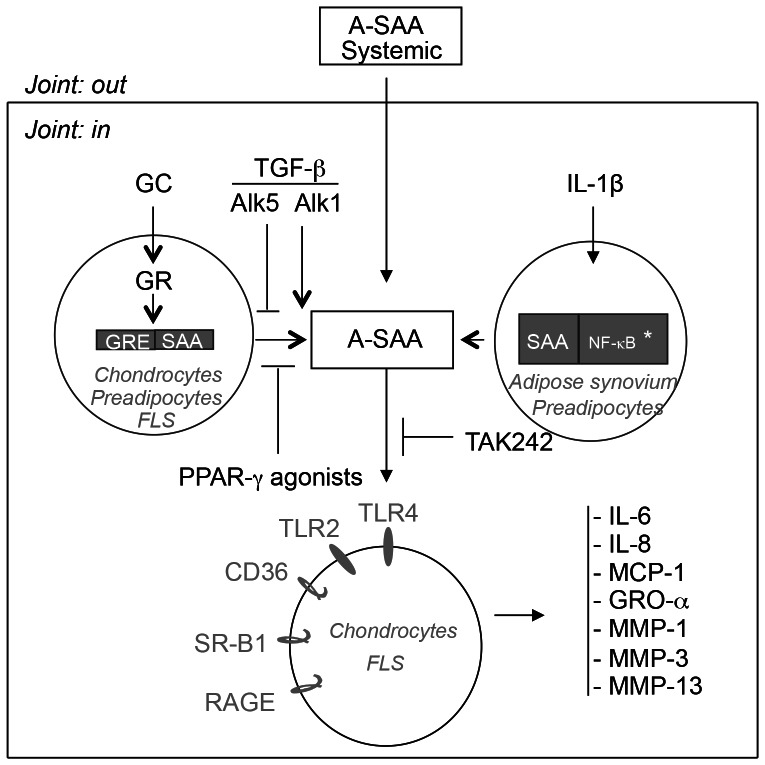
Origine and roles of A-SAA in joints. A-SAA is probably mainly due to the passive diffusion process from blood into the joint cavity. However, A-SAA expression in joints may also arise from primary chondrocytes, FLS and preadipocytes under glucocorticoid treatment, from dedifferentiated chondrocytes under spontaneous release and from preadipocytes and adipose synovium under IL-1β treatment. Glucocorticoid-induced A-SAA expression is GR-dependent and can be downregulated in the presence of PPAR-γ agonists (genistein and rosiglitazone) or under the control of TGF-β, with ALK1 as a stimulatory pathway and ALK5 as an inhibitory pathway. Spontaneous secretion of A-SAA by dedifferentiated chondrocytes can also be inhibited by TGF-β. TLR4 can mediate A-SAA-induced cytokines and MMPs expression in both primary chondrocytes and FLS, and be selectively inhibited by TAK242. * Ref [57].

We can summarize the data obtained as follows (Figure 7):

Several evidences strongly suggest that A-SAA in the synovial fluid of OA and RA patients could be mainly due to an increased level of A-SAA in blood and therefore due to the diffusion process into the joint cavity. Correlation between the Kellgren & Lauwrence grades and the synovial fluid (or plasma) levels of A-SAA support a role in the pathophysiological process of OA.

We have also demonstrated that A-SAA could be secreted by chondrocytes and FLS but mostly under glucocorticoids treatment. Cytokines (TNF-α, IL-1β and IL-6) or other mediators (LPS, leptin, insulin) did not induce any detectable A-SAA protein expression using both cell types. Spontaneous secretion was only observed using dedifferentiated chondrocytes and adipose synovium. Visceral preadipocytes produced A-SAA under glucocorticoid stimulation but also responded to cytokine stimuli.

A-SAA expression under glucocorticoid treatment involved GR but not MR receptor and was down-regulated by two PPAR-γ agonists (genistein and rosiglitazone). A-SAA production could be under the control of TGF-β, with ALK1 as a stimulatory pathway and ALK5 as an inhibitory pathway. Finally, rhSAA capacity of inducing cytokines and MMPs secretion by chondrocytes and FLS was restricted to TLR4 receptor.

## Supporting Information

Figure S1 **Human cytokines/MMP/TIMP antibody arrays.** Human primary chondrocytes and fibroblast-like synoviocytes (FLS) obtained from OA patients were cultured during 24 hours in the presence or absence of recombinant human A-SAA (5 µg/mL). Human cytokines/MMP/TIMP were detected in culture supernatants using antibody arrays.
(TIF)

Figure S2 **rhA-SAA inducing cytokines/MMPs/TIMPs expression.** A) OA primary chondrocytes and B) OA fibroblast-like synoviocytes after 24 hours of stimulation.
(TIF)

## References

[1] Dahaghin S, Bierma-Zeinstra SM, Koes BW, Hazes JM, Pols HA (2007) Do metabolic factors add to the effect of overweight on hand osteoarthritis? The Rotterdam Study. Ann Rheum Dis 66: 916–920. 17314121 10.1136/ard.2005.045724 PMC1955104

[2] Katz JD, Agrawal S, Velasquez M (2010) Getting to the heart of the matter: osteoarthritis takes its place as part of the metabolic syndrome. Curr Opin Rheumatol 22: 512–519. 20592604 10.1097/BOR.0b013e32833bfb4b

[3] Pottie P, Presle N, Terlain B, Netter P, Mainard D, et al (2006) Obesity and osteoarthritis: more complex than predicted! Ann Rheum Dis. 65: 1403–1405. 10.1136/ard.2006.061994 PMC1798356 17038451

[4] Puenpatom RA, Victor TW (2009) Increased prevalence of metabolic syndrome in individuals with osteoarthritis: an analysis of NHANES III data. Postgrad Med 121: 9–20. 19940413 10.3810/pgm.2009.11.2073

[5] Gomez R, Conde J, Scotece M, Gomez-Reino JJ, Lago F, et al (2011) What's new in our understanding of the role of adipokines in rheumatic diseases? Nat Rev Rheumatol 7: 528–536. 21808287 10.1038/nrrheum.2011.107

[6] Brennan FM, McInnes IB (2008) Evidence that cytokines play a role in rheumatoid arthritis. J Clin Invest 118: 3537–3545. 18982160 10.1172/JCI36389 PMC2575731

[7] Relic B, Zeddou M, Desoroux A, Beguin Y, de Seny D, et al (2009) Genistein induces adipogenesis but inhibits leptin induction in human synovial fibroblasts. Lab Invest 89: 811–822. 19434061 10.1038/labinvest.2009.41

[8] Conde J, Scotece M, Gomez R, Lopez V, Gomez-Reino JJ, et al (2011) Adipokines and osteoarthritis: novel molecules involved in the pathogenesis and progression of disease. Arthritis 2011: 203901. 22046513 10.1155/2011/203901 PMC3200120

[9] Yang RZ, Lee MJ, Hu H, Pollin TI, Ryan AS, et al (2006) Acute-phase serum amyloid A: an inflammatory adipokine and potential link between obesity and its metabolic complications. PLoS Med 3: e287. 16737350 10.1371/journal.pmed.0030287 PMC1472697

[10] Kushner I (1982) The phenomenon of the acute phase response. Ann N Y Acad Sci 389: 39–48. 7046585 10.1111/j.1749-6632.1982.tb22124.x

[11] Kushner I, Rzewnicki DL (1994) The acute phase response: general aspects. Baillieres Clin Rheumatol 8: 513–530. 7525083 10.1016/s0950-3579(05)80113-x

[12] Cai L, de Beer MC, de Beer FC, van der Westhuyzen DR (2005) Serum amyloid A is a ligand for scavenger receptor class B type I and inhibits high density lipoprotein binding and selective lipid uptake. J Biol Chem 280: 2954–2961. 15561721 10.1074/jbc.M411555200

[13] van der Westhuyzen DR, Cai L, de Beer MC, de Beer FC (2005) Serum amyloid A promotes cholesterol efflux mediated by scavenger receptor B-I. J Biol Chem 280: 35890–35895. 16120612 10.1074/jbc.M505685200

[14] Connolly M, Marrelli A, Blades M, McCormick J, Maderna P, et al (2010) Acute serum amyloid A induces migration, angiogenesis, and inflammation in synovial cells in vitro and in a human rheumatoid arthritis/SCID mouse chimera model. J Immunol 184: 6427–6437. 20435930 10.4049/jimmunol.0902941

[15] Mullan RH, Bresnihan B, Golden-Mason L, Markham T, O'Hara R, et al (2006) Acute-phase serum amyloid A stimulation of angiogenesis, leukocyte recruitment, and matrix degradation in rheumatoid arthritis through an NF-kappaB-dependent signal transduction pathway. Arthritis Rheum 54: 105–114. 16385502 10.1002/art.21518

[16] Koga T, Torigoshi T, Motokawa S, Miyashita T, Maeda Y, et al (2008) Serum amyloid A-induced IL-6 production by rheumatoid synoviocytes. FEBS Lett 582: 579–585. 18243142 10.1016/j.febslet.2008.01.022

[17] Connolly M, Mullan RH, McCormick J, Matthews C, Sullivan O, et al (2012) Acute-phase serum amyloid A regulates tumor necrosis factor alpha and matrix turnover and predicts disease progression in patients with inflammatory arthritis before and after biologic therapy. Arthritis Rheum 64: 1035–1045. 22076945 10.1002/art.33455

[18] Jousilahti P, Salomaa V, Rasi V, Vahtera E, Palosuo T (2001) The association of c-reactive protein, serum amyloid a and fibrinogen with prevalent coronary heart disease--baseline findings of the PAIS project. Atherosclerosis 156: 451–456. 11395043 10.1016/s0021-9150(00)00681-x

[19] Miida T, Yamada T, Seino U, Ito M, Fueki Y, et al (2006) Serum amyloid A (SAA)-induced remodeling of CSF-HDL. Biochim Biophys Acta 1761: 424–433. 16651021 10.1016/j.bbalip.2006.03.013

[20] O'Hara R, Murphy EP, Whitehead AS, FitzGerald O, Bresnihan B (2000) Acute-phase serum amyloid A production by rheumatoid arthritis synovial tissue. Arthritis Res 2: 142–144. 11062604 10.1186/ar78 PMC17807

[21] O'Hara R, Murphy EP, Whitehead AS, FitzGerald O, Bresnihan B (2004) Local expression of the serum amyloid A and formyl peptide receptor-like 1 genes in synovial tissue is associated with matrix metalloproteinase production in patients with inflammatory arthritis. Arthritis Rheum 50: 1788–1799. 15188355 10.1002/art.20301

[22] Cunnane G, Grehan S, Geoghegan S, McCormack C, Shields D, et al (2000) Serum amyloid A in the assessment of early inflammatory arthritis. J Rheumatol 27: 58–63. 10648018

[23] Vallon R, Freuler F, Desta-Tsedu N, Robeva A, Dawson J, et al (2001) Serum amyloid A (apoSAA) expression is up-regulated in rheumatoid arthritis and induces transcription of matrix metalloproteinases. J Immunol 166: 2801–2807. 11160347 10.4049/jimmunol.166.4.2801

[24] Altman R, Asch E, Bloch D, Bole G, Borenstein D, et al (1986) Development of criteria for the classification and reporting of osteoarthritis. Classification of osteoarthritis of the knee. Diagnostic and Therapeutic Criteria Committee of the American Rheumatism Association. Arthritis Rheum 29: 1039–1049. 3741515 10.1002/art.1780290816

[25] Arnett FC, Edworthy SM, Bloch DA, MacShane DJ, Fries JF, et al (1988) The American Rheumatism Association 1987 revised criteria for the classification of rheumatoid arthritis. Arthritis Rheum 31: 315–324. 3358796 10.1002/art.1780310302

[26] Kellgren JH, Lawrence JS (1957) Radiological assessment of osteo-arthrosis. Ann Rheum Dis 16: 494–502. 13498604 10.1136/ard.16.4.494 PMC1006995

[27] Relic B, Bentires-Alj M, Ribbens C, Franchimont N, Guerne PA, et al (2002) TNF-alpha protects human primary articular chondrocytes from nitric oxide-induced apoptosis via nuclear factor-kappaB. Lab Invest 82: 1661–1672. 12480916 10.1097/01.lab.0000041714.05322.c0

[28] Relic B, Benoit V, Franchimont N, Kaiser MJ, Hauzeur JP, et al (2006) Peroxisome proliferator-activated receptor-gamma1 is dephosphorylated and degraded during BAY 11-7085-induced synovial fibroblast apoptosis. J Biol Chem 281: 22597–22604. 16766531 10.1074/jbc.M512807200

[29] Wentworth JM, Naselli G, Brown WA, Doyle L, Phipson B, et al (2010) Pro-inflammatory CD11c+CD206+ adipose tissue macrophages are associated with insulin resistance in human obesity. Diabetes 59: 1648–1656. 20357360 10.2337/db09-0287 PMC2889764

[30] Emi N, Friedmann T, Yee JK (1991) Pseudotype formation of murine leukemia virus with the G protein of vesicular stomatitis virus. J Virol 65: 1202–1207. 1847450 10.1128/jvi.65.3.1202-1207.1991 PMC239887

[31] Benya PD, Padilla SR, Nimni ME (1978) Independent regulation of collagen types by chondrocytes during the loss of differentiated function in culture. Cell 15: 1313–1321. 729001 10.1016/0092-8674(78)90056-9

[32] von der Mark K, Gauss V, von der Mark H, Muller P (1977) Relationship between cell shape and type of collagen synthesised as chondrocytes lose their cartilage phenotype in culture. Nature 267: 531–532. 559947 10.1038/267531a0

[33] Blaney Davidson EN, Remst DF, Vitters EL, van Beuningen HM, Blom AB, et al (2009) Increase in ALK1/ALK5 ratio as a cause for elevated MMP-13 expression in osteoarthritis in humans and mice. J Immunol 182: 7937–7945. 19494318 10.4049/jimmunol.0803991

[34] van den Berg WB (2011) Osteoarthritis year 2010 in review: pathomechanisms. Osteoarthritis Cartilage 19: 338–341. 21324370 10.1016/j.joca.2011.01.022

[35] Lee MS, Yoo SA, Cho CS, Suh PG, Kim WU, et al (2006) Serum amyloid A binding to formyl peptide receptor-like 1 induces synovial hyperplasia and angiogenesis. J Immunol 177: 5585–5594. 17015746 10.4049/jimmunol.177.8.5585

[36] Mullan RH, McCormick J, Connolly M, Bresnihan B, Veale DJ, et al (2010) A role for the high-density lipoprotein receptor SR-B1 in synovial inflammation via serum amyloid-A. Am J Pathol 176: 1999–2008. 20304957 10.2353/ajpath.2010.090014 PMC2843487

[37] Baranova IN, Bocharov AV, Vishnyakova TG, Kurlander R, Chen Z, et al (2010) CD36 is a novel serum amyloid A (SAA) receptor mediating SAA binding and SAA-induced signaling in human and rodent cells. J Biol Chem 285: 8492–8506. 20075072 10.1074/jbc.M109.007526 PMC2832998

[38] Cheng N, He R, Tian J, Ye PP, Ye RD (2008) Cutting edge: TLR2 is a functional receptor for acute-phase serum amyloid A. . J Immunol 181: 22–26. 18566366 10.4049/jimmunol.181.1.22 PMC2464454

[39] Sandri S, Rodriguez D, Gomes E, Monteiro HP, Russo M, et al (2008) Is serum amyloid A an endogenous TLR4 agonist? J Leukoc Biol 83: 1174–1180. 18252871 10.1189/jlb.0407203

[40] Cai H, Song C, Endoh I, Goyette J, Jessup W, et al (2007) Serum amyloid A induces monocyte tissue factor. J Immunol 178: 1852–1860. 17237436 10.4049/jimmunol.178.3.1852

[41] Okamoto H, Katagiri Y, Kiire A, Momohara S, Kamatani N (2008) Serum amyloid A activates nuclear factor-kappaB in rheumatoid synovial fibroblasts through binding to receptor of advanced glycation end-products. J Rheumatol 35: 752–756. 18322992

[42] Bozinovski S, Uddin M, Vlahos R, Thompson M, McQualter JL, et al (2012) Serum amyloid A opposes lipoxin A(4) to mediate glucocorticoid refractory lung inflammation in chronic obstructive pulmonary disease. Proc Natl Acad Sci U S A 109: 935–940. 22215599 10.1073/pnas.1109382109 PMC3271884

[43] Matsunaga N, Tsuchimori N, Matsumoto T, Ii M (2011) TAK-242 (resatorvid), a small-molecule inhibitor of Toll-like receptor (TLR) 4 signaling, binds selectively to TLR4 and interferes with interactions between TLR4 and its adaptor molecules. Mol Pharmacol 79: 34–41. 20881006 10.1124/mol.110.068064

[44] de Seny D, Fillet M, Ribbens C, Maree R, Meuwis MA, et al (2008) Monomeric calgranulins measured by SELDI-TOF mass spectrometry and calprotectin measured by ELISA as biomarkers in arthritis. Clin Chem 54: 1066–1075. 18436720 10.1373/clinchem.2007.099549

[45] Oliviero F, Lo Nigro A, Bernardi D, Giunco S, Baldo G, et al (2012) A comparative study of serum and synovial fluid lipoprotein levels in patients with various arthritides. Clin Chim Acta 413: 303–307. 22037510 10.1016/j.cca.2011.10.019

[46] Stokes DG, Liu G, Dharmavaram R, Hawkins D, Piera-Velazquez S, et al (2001) Regulation of type-II collagen gene expression during human chondrocyte de-differentiation and recovery of chondrocyte-specific phenotype in culture involves Sry-type high-mobility-group box (SOX) transcription factors. Biochem J 360: 461–470. 11716775 10.1042/0264-6021:3600461 PMC1222247

[47] Hayes AJ, Hall A, Brown L, Tubo R, Caterson B (2007) Macromolecular organization and in vitro growth characteristics of scaffold-free neocartilage grafts. J Histochem Cytochem 55: 853–866. 17478447 10.1369/jhc.7A7210.2007

[48] Fukui N, Ikeda Y, Tanaka N, Wake M, Yamaguchi T, et al (2011) alphavbeta5 integrin promotes dedifferentiation of monolayer-cultured articular chondrocytes. Arthritis Rheum 63: 1938–1949. 21425129 10.1002/art.30351

[49] Yoon YM, Kim SJ, Oh CD, Ju JW, Song WK, et al (2002) Maintenance of differentiated phenotype of articular chondrocytes by protein kinase C and extracellular signal-regulated protein kinase. J Biol Chem 277: 8412–8420. 11744731 10.1074/jbc.M110608200

[50] Thorn CF, Lu ZY, Whitehead AS (2004) Regulation of the human acute phase serum amyloid A genes by tumour necrosis factor-alpha, interleukin-6 and glucocorticoids in hepatic and epithelial cell lines. Scand J Immunol 59: 152–158. 14871291 10.1111/j.0300-9475.2004.01369.x

[51] Thorn CF, Whitehead AS (2002) Differential glucocorticoid enhancement of the cytokine-driven transcriptional activation of the human acute phase serum amyloid A genes, SAA1 and SAA2. J Immunol 169: 399–406. 12077270 10.4049/jimmunol.169.1.399

[52] Kumon Y, Suehiro T, Faulkes DJ, Hosakawa T, Ikeda Y, et al (2002) Transcriptional regulation of serum amyloid A1 gene expression in human aortic smooth muscle cells involves CCAAT/enhancer binding proteins (C/EBP) and is distinct from HepG2 cells. Scand J Immunol 56: 504–511. 12410800 10.1046/j.1365-3083.2002.01169.x

[53] Ganapathi MK, Rzewnicki D, Samols D, Jiang SL, Kushner I (1991) Effect of combinations of cytokines and hormones on synthesis of serum amyloid A and C-reactive protein in Hep 3B cells. J Immunol 147: 1261–1265. 1651357

[54] Conde J, Gomez R, Bianco G, Scotece M, Lear P, et al (2011) Expanding the adipokine network in cartilage: identification and regulation of novel factors in human and murine chondrocytes. Ann Rheum Dis 70: 551–559. 21216818 10.1136/ard.2010.132399

[55] Zeddou M, Relic B, Malaise O, Charlier E, Desoroux A, et al (2012) Differential Signalling Through ALK-1 and ALK-5 Regulates Leptin Expression in Mesenchymal Stem Cells. Stem Cells Dev 21: 1948–1955. 22087763 10.1089/scd.2011.0321 PMC3396155

[56] Nair A, Kanda V, Bush-Joseph C, Verma N, Chubinskaya S, et al (2012) Synovial fluid from patients with early osteoarthritis modulates fibroblast-like synoviocyte responses to toll-like receptor 4 and toll-like receptor 2 ligands via soluble CD14. Arthritis Rheum 64: 2268–2277. 22492243 10.1002/art.34495 PMC3386375

[57] Vlasova MA, Moshkovskii SA (2006) Molecular interactions of acute phase serum amyloid A: possible involvement in carcinogenesis. Biochemistry (Mosc) 71: 1051–1059. 17125452 10.1134/s0006297906100014

