# Supplementary figures and images for: Acute-Phase Serum Amyloid A in Osteoarthritis: Regulatory Mechanism and Proinflammatory Properties
Source: PLoS One. 2013 Jun 12;8(6):e66769. doi: 10.1371/journal.pone.0066769 (PMC3680431; doi:10.1371/journal.pone.0066769)

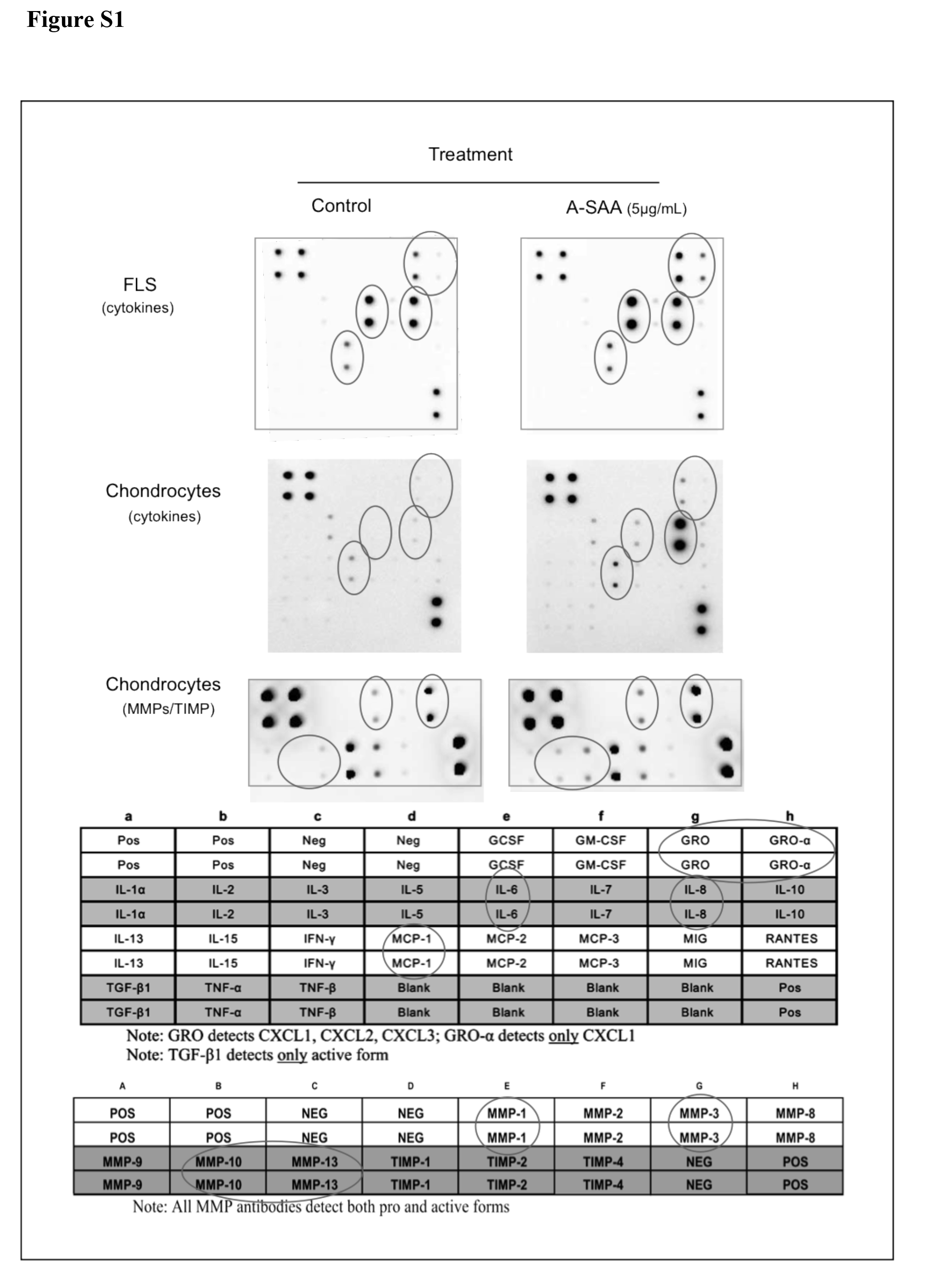

Supplement: Figure S1 — Human cytokines/MMP/TIMP antibody arrays. Human primary chondrocytes and fibroblast-like synoviocytes (FLS) obtained from OA patients were cultured during 24 hours in the presence or absence of recombinant human A-SAA (5 µg/mL). Human cytokines/MMP/TIMP were detected in culture supernatants using antibody arrays. (TIF) [file pone.0066769.s001.tif]

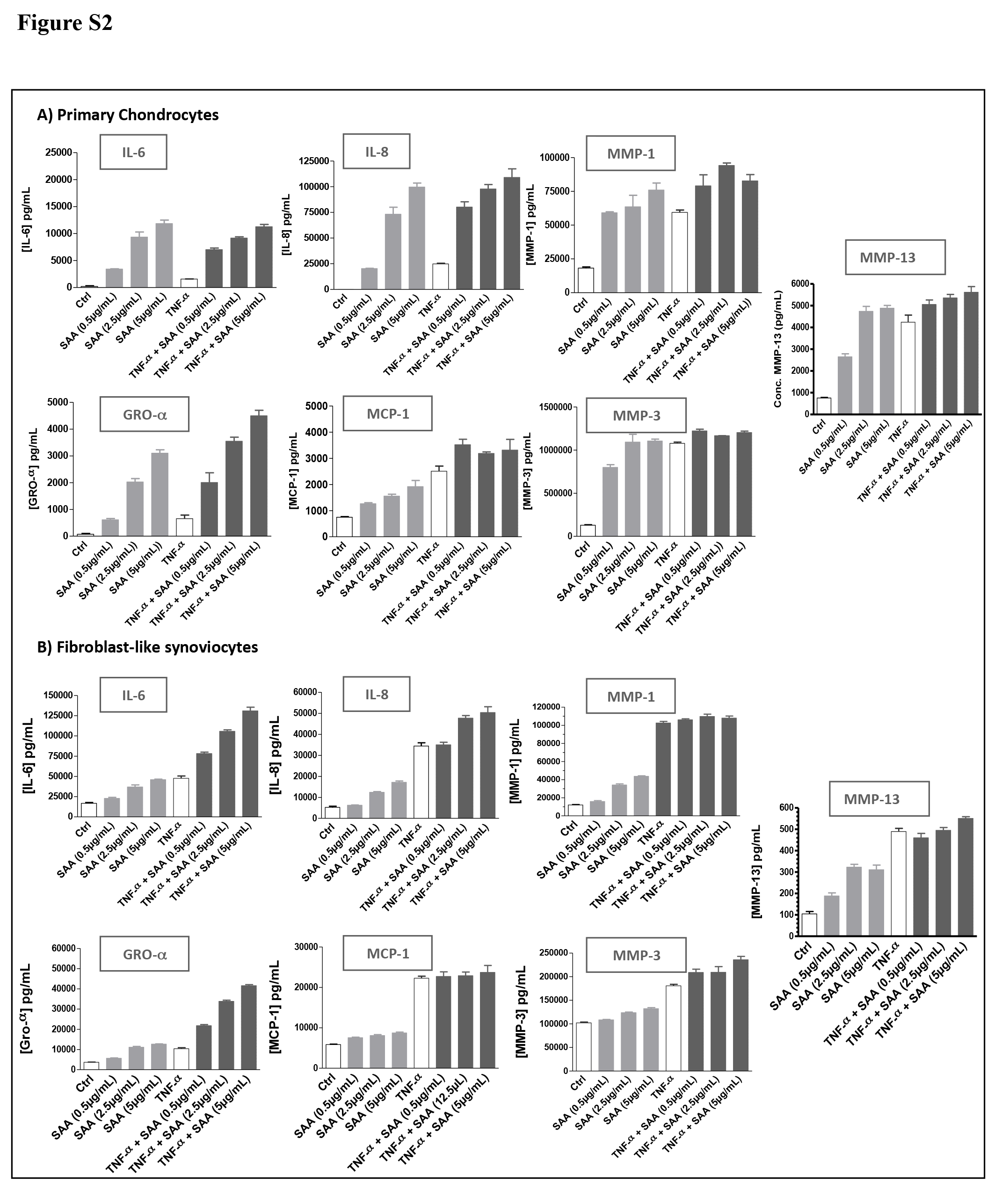

Supplement: Figure S2 — rhA-SAA inducing cytokines/MMPs/TIMPs expression. A) OA primary chondrocytes and B) OA fibroblast-like synoviocytes after 24 hours of stimulation. (TIF) [file pone.0066769.s002.tif]
